# Methods to Measure the Impact of Home, Social, and Sexual Neighborhoods of Urban Gay, Bisexual, and Other Men Who Have Sex with Men

**DOI:** 10.1371/journal.pone.0075878

**Published:** 2013-10-16

**Authors:** Beryl A. Koblin, James E. Egan, Andrew Rundle, James Quinn, Hong-Van Tieu, Magdalena Cerdá, Danielle C. Ompad, Emily Greene, Donald R. Hoover, Victoria Frye

**Affiliations:** 1 Laboratory of Infectious Disease Prevention, Lindsley F. Kimball Research Institute, New York Blood Center, New York, New York, United States of America; 2 Department of Behavioral and Community Health Sciences, Graduate School of Public Health, University of Pittsburgh, Pittsburgh, Pennsylvania, United States of America; 3 Department of Epidemiology, Mailman School of Public Health, Columbia University, New York, New York, United States of America; 4 Division of Infectious Diseases, Department of Medicine, Columbia University College of Physicians and Surgeons, New York, New York, United States of America; 5 Center for Health, Identity, Behavior, and Prevention Studies (CHIBPS) and Department of Nutrition, Food Studies and Public Health, Steinhardt School of Culture, Education and Human Development, New York University, New York, New York, United States of America; 6 Laboratory of Social and Behavioral Sciences, Lindsley F. Kimball Research Institute, New York Blood Center, New York, New York, United States of America; 7 Department of Statistics and Biostatistics and Institute for Health, Health Care Policy and Aging Research, Rutgers, The State University of New Jersey, Piscataway, New Jersey, United States of America; 8 Department of Sociomedical Sciences, Mailman School of Public Health, Columbia University, New York, New York, United States of America; Rollins School of Public Health, Emory University, United States of America

## Abstract

Men who have sex with men (MSM) accounted for 61% of new HIV diagnoses in the United States in 2010. Recent analyses indicate that socio-structural factors are important correlates of HIV infection. NYCM2M was a cross-sectional study designed to identify neighborhood-level characteristics within the urban environment that influence sexual risk behaviors, substance use and depression among MSM living in New York City. The sample was recruited using a modified venue-based time-space sampling methodology and through select websites and mobile applications.

This paper describes novel methodological approaches used to improve the quality of data collected for analysis of the impact of neighborhoods on MSM health. Previous research has focused predominately on residential neighborhoods and used pre-determined administrative boundaries (e.g., census tracts) that often do not reflect authentic and meaningful neighborhoods. This study included the definition and assessment of multiple neighborhoods of influence including where men live (home neighborhood), socialize (social neighborhood) and have sex (sexual neighborhood). Furthermore, making use of technological advances in mapping, we collected geo-points of reference for each type of neighborhood and identified and constructed self-identified neighborhood boundary definitions. Finally, this study collected both perceived neighborhood characteristics and objective neighborhood conditions to create a comprehensive, flexible and rich neighborhood-level set of covariates. This research revealed that men perceived their home, social and sexual neighborhoods in different ways. Few men (15%) had the same home, social and sexual neighborhoods; for 31%, none of the neighborhoods was the same. Of the three types of neighborhoods, the number of unique social neighborhoods was the lowest; the size of sexual neighborhoods was the smallest. The resultant dataset offers the opportunity to conduct analyses that will yield context-specific and nuanced understandings of the relations among neighborhood space, and the well-being and health of urban MSM.

## Introduction

In 2010, men who have sex with men (MSM) accounted for 61% of new HIV diagnoses in the United States (US) [1]. Despite decreasing trends in HIV diagnoses attributable to either injection drug use or heterosexual contact, new diagnoses among MSM generally, and among young (ages 13–29), Black MSM in particular, increased during 2007–2010 [1]. A recent meta-analysis of 164 studies ranked correlates of HIV infection among MSM and found that socio-structural factors constituted the majority of the top 10 correlates, including low income and education, recent unemployment, and history of incarceration, with Black MSM more likely to experience these factors than MSM of other races/ethnicities [2]. These results demonstrate the potential important role of the social environment in HIV infection risk among MSM.

A substantial body of literature describes the impact of the social and physical environment, as constituted in neighborhood conditions, on individual and community health and well-being [3]-[8]. Frye and colleagues [9] assessed how researchers have explored the roles of the physical, social, and cultural environments in sexual health outcomes predominantly among heterosexuals and found that studies have generally been framed within theories of physical disorder [10], social disorganization [11]–[13] and social norms and influence [14]–[16]. For example, neighborhoods with greater poverty and disorder have been found to have higher rates of sexually transmitted infections (STIs) [10]. However, little is known about how neighborhood may have an impact on sexual risk, mental health and well-being specifically among MSM. In understanding the urban environment and health outcomes among MSM, the lives of MSM are likely shaped by factors that overlap with heterosexuals and factors that are unique to MSM. Thus, Frye et al [9] drew on existing neighborhood effects theories as mentioned above, but also integrated social identity [17], [18] and sexual minority stress [19] theories into a socioecological framework to describe potential relations (both positive and negative) among identity, the environment and MSM sexual behavior and health.

A few recent studies suggest that neighborhoods have an important impact on the socio-sexual lives of urban MSM. In a qualitative study [20], [21], we reported that urban MSM identified boundaries of their home, social, and places of sex (labeled as “sexual”) neighborhoods, and described both a sense of connection with each as well as their perceptions of the interaction between these spaces and their sexual behaviors and health. Other studies have found that neighborhood gay presence (i.e., proportion of same sex-headed households) was associated with differences in community involvement, friend and sexual partner selection [22], consistent condom use and increased HIV testing [22]–[24] but also increased use of specific substances, such as methamphetamines [25], [26]. In contrast, Buttram and Kurtz found a higher level of unprotected sex and a lower level of social engagement but lower levels of cocaine use and substance dependence were associated with gay neighborhood residence [26]. Jones et al. found that MSM residing in neighborhoods with greater residential mobility were more likely to report being HIV-negative, have greater HIV knowledge, and participate in prevention activities. In addition, participation in HIV prevention activities was enhanced in neighborhoods of predominately female-headed households and lessened in economically depressed areas [24].

These studies, which provided preliminary evidence of the impact of neighborhood on the lives of MSM, possess several methodological limitations that often characterize neighborhood effects research. First, a persistent issue in neighborhood research is how to define “neighborhoods”. The size and definition of the geographic area that matters for health depends on the pathways through which the neighborhood effect is hypothesized to operate and the outcomes being studied [27]. Most studies have used geopolitical boundaries such as counties, census tracts or zip codes, which do not necessarily correspond to the theoretically relevant geographic area of interest, often do not resonate with study participants as neighborhoods and constrain the level of analysis possible [28], [29]. Recent discussions of advances in neighborhood effects research methodology urge analysts to use spatial approaches that allow the construction of unique buffers around residences in order to better reflect the variable influence of environmental factors [30]. Second, despite recognition that health is defined by multiple contexts that individuals inhabit, including home and social environment, the bulk of neighborhood research has only focused on residential neighborhood. Finally, data on neighborhood characteristics that are particularly relevant to sub-populations, such as MSM, are often not publicly available and thus important neighborhood-level covariates, such as neighborhood-level homophobia and sexual orientation-based discrimination, are not available for analyses.

The NYCM2M study was a cross-sectional study designed to identify neighborhood-level characteristics within the urban environment associated with sexual risk behaviors, substance use and depression among MSM living in New York City (NYC). The study was designed to minimize the limitations of previous work and use spatial methods to allow MSM to identify the location of multiple neighborhoods that can matter to their sexual health, including neighborhoods where they reside, socialize and engage in sex and to define the neighborhood scales that were most relevant to them. The purpose of this paper is to describe the methodology used to identify and describe home, social and sexual neighborhoods of MSM in an urban environment.

## Methods

### Ethics Statement

The New York Blood Center Institutional Review Board first approved this study and provides on-going oversight. Subsequently, institutional review boards at co-investigator institutions including New York University University Committee on Activities Involving Human Subjects, Hunter College Institutional Review Board and the New York Academy of Medicine Institutional Review Board also reviewed the study. Columbia University Institutional Review Board designated the study as non-research as the data processing contributions of Rundle and Quinn were not considered to be human subjects research. All participants completed written informed consent. All data collected, other than HIV testing, were gathered by participant self-report using audio computer-assisted self-interview (ACASI) technology and interviewers.

### Study Sample and Recruitment

The study’s recruitment priority was to obtain a study sample from as many different neighborhoods in NYC using a systematic sampling method. Thus, MSM were recruited using a modified venue-based time-space sampling methodology and through banner ads on select websites [31]. A sample of physical locations and associated day-time periods were randomly selected each month from a sampling frame. The sampling frame included a wide range of neighborhoods across the NYC boroughs that are traditionally considered gay enclaves, those with a growing gay population, as well as neighborhoods with a much less visible or documented gay presence. Locations within neighborhoods included street locations, retail businesses, and bars and clubs. An internet- and mobile application-based recruitment strategy was added in July 2012 in response to the proliferation of internet-based networking applications, in particular geosocial networking applications, which appeared since the start of recruitment. We selected three types of websites and mobile applications to expand the reach of recruitment to additional neighborhoods. First, we placed banner ads on BGClive.com, a website focused on Black and Latino MSM. Second, we placed ads on Facebook.com with the focus of mass distribution in NYC. Finally, we utilized Grindr.com, a geosocial networking application geared towards MSM. The ads were placed approximately three months apart.

Recruitment occurred at the locations during designated sampling events. Men were systematically approached (e.g., every third man) at the sampling events and screened for preliminary eligibility. Eligible participants were invited to provide contact information. A similar process occurred for website and mobile application recruitment as men were directed to the NYCM2M website, screened for preliminary eligibility, and those eligible were asked to provide contact information. Attempts were made to contact all potential participants to screen for eligibility and schedule a study visit.

Individuals were eligible to participate if they report being a biological male at birth, were at least 18 years of age, resided in NYC, reported engaging in anal sex with a man in the past 3 months, communicated in English or Spanish and were willing and able to give informed consent for the study.

### Study visit

Recruited men were given the choice between two study sites, one located at Union Square in lower Manhattan and one in the South Bronx (added July 2012 to increase participation of men in outer boroughs). After providing informed consent, participants met with a staff member to complete the Neighborhood Locator Questionnaire which collected information on the location of four neighborhoods: home (where they live), social (where they socialize most often) and sexual (where they most recently had sex and most often have sex) (see below for details), as well as place of birth and place where the majority of their childhood was spent. Participants then completed an assessment using ACASI technology. Following completion of the ACASI assessment, a social and sexual network questionnaire was completed with an interviewer with data entry into a computer system. Participants then received HIV risk-reduction counseling and a rapid HIV antibody test was conducted. If the rapid HIV test was reactive, HIV infection was confirmed by Western Blot testing. Participants with HIV infection (newly diagnosed and previous known infection) were asked to provide a blood sample to test for CD4 cell count and HIV viral load. Participants testing HIV positive were referred for treatment and medical and social services, as needed. Upon completion of the visit, participants received $50 and a two-way Metrocard for their time and transportation costs.

### Measures: Individual and situational measures

Individual-level measures used in this analysis included demographics, general and HIV-related health questions (e.g. HIV testing history, occurrence of STIs), history of incarceration and sexual identity. Sexual behaviors in the three months prior to the study included number of partners, number of insertive and receptive anal sex acts and use of condoms and partner HIV status. Substance use questions included frequency of use in the past three months.

### Measures: Neighborhood Location and Boundaries

To inform the design of the neighborhood assessment component of this study, we conducted formative qualitative research, described elsewhere [20], to assess how MSM conceptualize their neighborhoods and to explore the meaning of neighborhood boundaries, types and characteristics among participants, as well as their perceptions of the impact of neighborhood characteristics on their health, well-being and sexual behavior. Results of this work revealed the need to assess self-defined neighborhood definitions for use in combination with pre-identified administrative boundaries (e.g. city or census defined boundaries) and pre-defined characteristics (e.g. poverty rates, crime rates, etc.). Our formative research also provided initial insights into neighborhood typologies and functions, allowing us to map neighborhoods falling into “home” “social” and “sexual” categories. Preliminary analyses explored the importance of neighborhood characteristics that may be generically important to residents, such as crime and cleanliness, and others that may have special importance for MSM, such as gay presence, homophobia, social norms, and tolerance. Similarly, the presence of same-race/ethnicity residents emerged as important to the men interviewed. The formative work confirmed that some men were more able to choose their neighborhoods and that this mobility often correlates with social class and social background [21].

Geographic neighborhood data were collected from each participant prior to the ACASI survey. Identification of home, social and sexual (most recent and most often) neighborhoods in the neighborhood locator questionnaire, mentioned above, was accomplished using Google Earth [32] to help participants identify specific locations from which we were then able to collect geospatial coordinates. This process is described below.

Participants were first asked to characterize their home neighborhood, defined as the neighborhood in which they were currently living, by identifying the borough (drop down list), neighborhood name (drop down list of 347 neighborhoods) and how long they have lived there. Next, interviewers assisted participants in using Google Earth to ‘drop a pin’ at the closest intersection near their home. The latitude and longitude of the pin drop were recorded using the Google Earth application providing a centroid location for the home neighborhood. All data were stored locally. To better understand perceived geographical neighborhoods, participants were then asked, “*When you think about your home neighborhood, what area do you usually think of*”. Response categories were: *the block you live on*, *the area within 5 blocks around the place you live*, *the area within 10 blocks around the place you live*, or *an area larger than 10 blocks around the place you live*. This process was then repeated for each neighborhood of interest including: Social Neighborhood: *Think of the neighborhood that you spent the most time socializing/hanging out in during the past 3 months?* Recent Sexual Neighborhood: *Think of the neighborhood that you most recently had sex?* Most Often Sexual Neighborhood: *Think of the neighborhood that you most often had sex in during the past 3 months?*

Several steps were taken to ensure ease of use and accurate data collection with Google Earth. This section of the survey was interviewer assisted; participants were walked through each step of using Google Earth from locating specific places to inserting their unique ‘pins’. Interviewers received extensive training on using Google Earth and in using urban cues to help participants remember the location of spaces (e.g. identifying the borough and then working with participants to identify landmarks, subway stops, or other spaces to help them choose specific locations). Study staff began by telling the participant that they would be placing a pin near the person’s home location. They also reassured the participant that the pin drop was placed at the closest intersection, and not directly on the person’s home. Study staff reports suggested that participants were not bothered by the process or concerned about providing the data. Participants were interested and engaged and even searched through old text messages or online profiles so as to more accurately identify locations.

### Measures: Self-reported Neighborhood Characteristics

Additional questions on the characteristics of the identified home and social neighborhoods were collected in the ACASI interview. These questions were designed to assess participants’ perceptions of these neighborhoods including such factors as numbers of friends and family living there, cohesion, engagement, and safety. We included both validated neighborhood assessment scales and, when appropriate, modified questions specific to the NYC and/or MSM context.

Data on duration and intensity of neighborhood exposure included measures on duration of residence, why they reside there, how long they plan on living there, home ownership, and how much time spent in each of the neighborhoods [33], [34]. Social ties were assessed by asking participants to identify the number and frequency of visits by friends and relatives in their home and social neighborhoods [35]. Social cohesion and trust were measured using a modified version of Sampson and Morenoff [36], including questions about respondents’ perceptions of connectedness and trust in their community [11], [36], [37]. Neighborhood involvement and social participation were assessed with questions related to involvement in local community or other groups (e.g., local neighborhood groups or block associations) and interaction with neighbors [11].

Perceived social disorder, physical decay, safety and overall neighborhood quality were assessed using the Ross and Mirowsky scale [38]. Safety questions from this instrument were adapted to reflect the unique safety concerns of MSM. Neighborhood integration, sense of community and identification with neighborhood were assessed using Perkin’s Sense of Community Scale [39]. Perceptions of a neighborhood’s characteristics, including external evaluation, general attachment, commitment and familiarity were assessed using the Urban Identity Scale [40]. Self-reported feeling of identification with neighborhood was assessed using the Three Dimensional Strength of Identification Scale [41] which included both cognitive and affective aspects of neighborhood identification and attachment as well as ties and feelings of similarity.

We measured three types of social norms. Substance use social norms were assessed using modified questions from the National Survey on Drug Use and Health [42] and covered constructs including criminalization, acceptability of substance use and awareness/tolerance of substance- using individuals. Perceived peer sexual behavior norms were assessed concerning multiple partners, safer sexual behaviors and condom use [43]. For use in analyses requiring neighborhood norms, we plan to aggregate individual responses to substance use social norms and perceived peer sexual behavior norms up to the relevant geographic unit. Finally, perceptions of homophobia and racial discrimination in home and social neighborhoods were assessed using an adapted version of Krieger's perceived discrimination scale [44].

### Measures: Objective Neighborhood Characteristics

An extensive database was used to characterize objective measures of demographic and socioeconomic conditions of NYC neighborhoods. Secondary data sources, including the US Census [45], the NYC Housing and Vacancy Survey (NYCHVS) [46] and the NYC Mayor’s Management Report [47], were used to characterize issues such as socioeconomic status, housing quality, ethnicity, residential stability, crime rates, and cleanliness of streets and sidewalks. Other secondary sources were used to determine further neighborhood characteristics including neighborhood safety (every presentation since 1996 to an emergency department resulting from an assault has been identified and geocoded); access to public transit (the location of every bus and subway stop in NYC); land use mix; location and quality of parks; green space (census of all the street trees in NYC); and location of commercial recreation facilities (information on 300,000+ businesses on NYC). Finally, we used geocoded data from the Medical Examiner’s Office of every unexpected death in NYC to measure neighborhood suicide and homicide rates. These archival neighborhood-level data were supplemented by both an in-person survey designed to measure neighborhood characteristics such as social cohesion and a database that uses a validated system for characterizing aspects of urban design (for example, transparency which describes whether pedestrians can perceive activity beyond a sidewalk and building line) using GIS measures created with ArcGIS and ArcView [48]–[50].

### Data Management and Analysis

Geographical data points representing the neighborhood centroids captured by Google Earth were exported as a KML file for analysis with ArcGIS (ArcGIS Desktop: Release 10. Redlands, CA: Environmental Systems Research Institute.). Neighborhood centroids and perceived neighborhood boundaries (self-perceived neighborhood size) were used to construct unique neighborhoods for each individual. These locations were linked to the survey data on sexual behaviors, physical and mental health indicators, drug and alcohol use, as well perceptions of neighborhood (e.g., social cohesion, homophobia, etc.) and archival data (e.g., Census and other administrative data) on neighborhood conditions and characteristics (e.g., concentrated poverty/affluence, racial segregation, physical disorder, etc.). These complementary datasets give a comprehensive view of the perceived and objective characteristics of NYC neighborhoods available for analysis. A variety of analytic methods will be utilized, as appropriate, such as spatial analysis, multi-level analysis and generalized estimating equations to estimate robust standard errors to account for within-small-area clustering of responses.

### Current Analysis

For this analysis, descriptive statistics of the study sample data were generated with frequency distributions for categorical measures and the means, standard deviations, medians and interquartile ranges for continuous measures. US Census data were retrieved from the NYC Department of City Planning website [51]. Data management and analysis were conducted using SAS software (SAS software version 9.3, 2010, SAS Institute Inc. Cary, NC, USA).

Building from the work of Gates [52], we calculated same sex headed-households for each NYC neighborhood tabulation areas (NTAs) which are census tract aggregations to the level of neighborhood or multiple neighborhoods currently used by the NYC Department of City Planning [46] using 2010 US census tract data. We created a shading scheme to reflect numbers of Census reported same sex headed-household by NYC neighborhood and then plotted self-reported home “pin drops” to provide a visual assessment of participants’ home neighborhood in relation to same sex headed-households. All pin drops were randomly jittered within census tract so as not to identify the exact location of the participant.

## Results

### Sociodemographics

The following is a “data snapshot” of 706 men recruited through venue-based sampling from October 2010 through July 2012. The average age was 32.4 (SD = 10.8); 34% of the sample was white (non-Hispanic); 31% Hispanic; 23% Black/African American and 11% reported another ethnicity (Table 1). Just under one-third (31%) were born in NYC. Over 50% of men reported possessing at least a college degree. Nearly a quarter of men (24%) reported an average personal income of less than $10,000 per year, 41% reported an income of $10,000-39,000 and 35% reported an income of $40,000 or greater. Approximately 5% of men reported a lifetime history of incarceration.

**Table 1:** Socio-demographics, sexual behaviors and drug use, NYC MSM, 2010 – 2012 (N = 706).

		N (%)
Age	Mean (SD)	32.4 (10.8)
	18–24	191 (27.1)
	25–29	183 (25.9)
	30–39	153 (21.7)
	40+	179 (25.4)
Race/ethnicity	White, Non-Hispanic	243 (34.4)
	Hispanic	218 (30.9)
	Black, Non-Hispanic	165 (23.4)
	Multiracial	29 (4.1)
	Asian/Pacific Islander	20 (2.9)
	Other/Refused to answer	31 (4.4)
NYC Born	Yes	220 (31.2)
	No	320 (45.3)
	Outside US/Puerto Rico	166 (23.5)
Education	< High school graduate	50 (7.1)
	High school graduate	87 (12.3)
	Some college	210 (29.8)
	College graduate or more	359 (50.9)
Annual personal income	< $10,000	164 (23.8)
	$10,000–$39,999	281 (40.8)
	$40,000–$59,999	114 (16.6)
	≥ $60,000	129 (18.8)
Past incarceration (ever)	Yes	38 (5.4)
Sexual identity	Homosexual/gay	615 (87.1)
	Bisexual	65 (9.2)
	Heterosexual/other	26 (3.7)
Currently married/registered domestic partner with a man	Yes	37 (5.2)
HIV serostatus	Positive (self-report)	152 (21.5)
	Positive (newly diagnosed)	9 (1.3)
	Negative (tested)	388 (55)
	Refused test (negative self report)	138 (19.5)
	Refused test (unknown self report)	18 (2.5)
	Unknown	1 (0.1)
Number sexual partners	Mean (SD), Median (IQR)	4.6 (7.3), 3 (1,5)
Sexual risk behavior in past 3 months^1^	UIA	144 (20.4)
	URA	129 (18.3)
	SDUIA	106 (15.0)
	SDURA	95 (13.5)
Substance Use in past 3 months	Heavy alcohol use^2^	99 (14.1)
	Marijuana	370 (52.6)
	Inhaled nitrites/poppers	229 (32.5)
	Powdered cocaine	146 (20.7)
	Crack cocaine	22 (3.1)
	Methamphetamine/amphetamine	33 (4.7)
	Club drugs (Special K, GHB, etc.)	77 (10.9)
	Erectile dysfunction medication	82 (11.7)
	Other Opiates/Benzodiazepines	38 (5.4)
STI in past 12 months	Yes	52 (7.4)

1 UIA  =  unprotected insertive anal intercourse; URA =  unprotected receptive anal intercourse; SDUIA: UIA with serodiscordant or unknown status partners; SDURA: URA with serodiscordant or unknown status partners;

2 4 or more times a week/3 or more drinks.

The majority of men (87%) self-identified as exclusively gay or homosexual; 9% self-identified as exclusively bisexual and 4% identified as straight, heterosexual or another identity. Only 5% of men sampled reported being married or in a registered domestic partnership with another man. Fifty-five percent of participants tested HIV negative, 22% self-reported as HIV-positive (of whom, 41% refused rapid HIV testing), 1% were considered newly diagnosed with HIV, and 22% refused HIV testing and self-reported being HIV- negative or of unknown status on ACASI (Table 1). One man had an unknown HIV status as conflicting test and self-report could not be resolved. The mean number of sex partners in the past 3 months was 4.6 (SD = 7.3) and the median was 3 (IQR =  1, 5). In terms of sexual HIV risk behavior, 15% of men reported unprotected insertive anal intercourse with HIV-discordant or unknown status partners and 14% of men reported unprotected receptive anal intercourse with HIV-discordant or unknown status partners (Table 1). In terms of drug and alcohol use in the past three months, 14% reported heavy drinking, 53% of men reported using marijuana; 33% inhaled nitrates or poppers, 21% powder cocaine, 13% opiates or benzodiazepines, 12% erectile dysfunction medication, 12% club drugs (Ketamine, GHB, MDMA, etc.), 5% methamphetamine and 3% crack cocaine. Less than 1 in 10 (7%) of men reported having a sexually transmitted infection in the past 12 months.

### Neighborhoods

The men were recruited from a wide range of neighborhoods in NYC. Thirty-seven percent lived in Manhattan; 35% in Brooklyn; 14% in Queens; 12% on the Bronx and 2% in Staten Island. We assessed the extent to which the sample accrued reflected the underlying population of men aged 18 and older and living in each of the NYC boroughs based on US Census data. The study sample over represented men residing in Manhattan (NYCM2M: 37% vs. US Census: 21%) and Brooklyn (NYCM2M: 35% vs. US Census: 29%) and underrepresented men from Queens (NYCM2M: 14% vs. US Census: 28%), the Bronx (NYCM2M: 12% vs. US Census: 15%) and Staten Island (NYCM2M: 2% vs. 6%).

**Home Neighborhood.** We recruited at least one resident from 147 of the 347 area neighborhoods and 144 of the 195 NTAs according to self-report neighborhoods and 151 of 195 NTAs according to “pin drop” Google Earth data. Twenty-two percent of men defined their home neighborhood as the block that they lived on; 27% reported that it was the area within 5 blocks of their home; 25% the area within 10 blocks; and 26% reported that it was an area larger than 10 blocks around their home (Table 2). The majority of men did not have relatives living in their home neighborhoods. In contrast, 71% reported having friends living in their home neighborhoods and 43% reported having any sex partners who lived in their home neighborhoods. When asked, 63% of the men reported that they would not live in their current home neighborhood if given the opportunity to live somewhere else in NYC.

**Table 2:** Neighborhood characteristics, NYC MSM, 2010 – 2012 (N = 706).

		Neighborhoods			
		Home	Social	Most often sex	Most recent sex
		N (%)	N (%)	N (%)	N (%)
Unique neighborhoods^1^		143 (41.2)	89 (25.7)	127 (36.6)	127 (36.6)
Boundary 2	1 block	154 (21.8)	101 (14.3)	404 (58.6)	421 (61.6)
	5 blocks	192 (27.2)	138 (19.6)	97 (14.1)	91 (13.3)
	10 blocks	178 (25.2)	216 (30.6)	92 (13.4)	76 (11.1)
	+ 10 blocks	182 (25.8)	250 (35.5)	96 (13.9)	95 (13.9)
No. of relatives in neighborhood^2^	0	525 (74.5)	613 (87.0)	--	--
	1-2	83 (11.8)	49 (7.0)		
	3-5	61 (8.7)	30 (4.3)		
	6+	36 (5.1)	13 (1.8)		
No. of friends in neighborhood^2^	0	206 (29.2)	216 (30.8)	--	--
	1-2	241 (34.1)	190 (27.1)		
	3-5	163 (23.1)	169 (24.1)		
	6-9	96 (13.6)	127(18.1)		
No. of sex partners in neighborhood^2^	0	403 (57.4)	421 (60.3)	--	--
	1-2	231 (32.9)	185 (26.5)		
	3-5	52 (7.4)	71 (10.2)		
	6+	16 (2.3)	21 (3.0)		

Note: Some of the N’s do not equal the total due to missing/not applicable responses.

1 Percent is of 347 neighborhoods listed in drop down list.

2 Percent is of 706 participants.

-- not asked.

**Figure 1:**
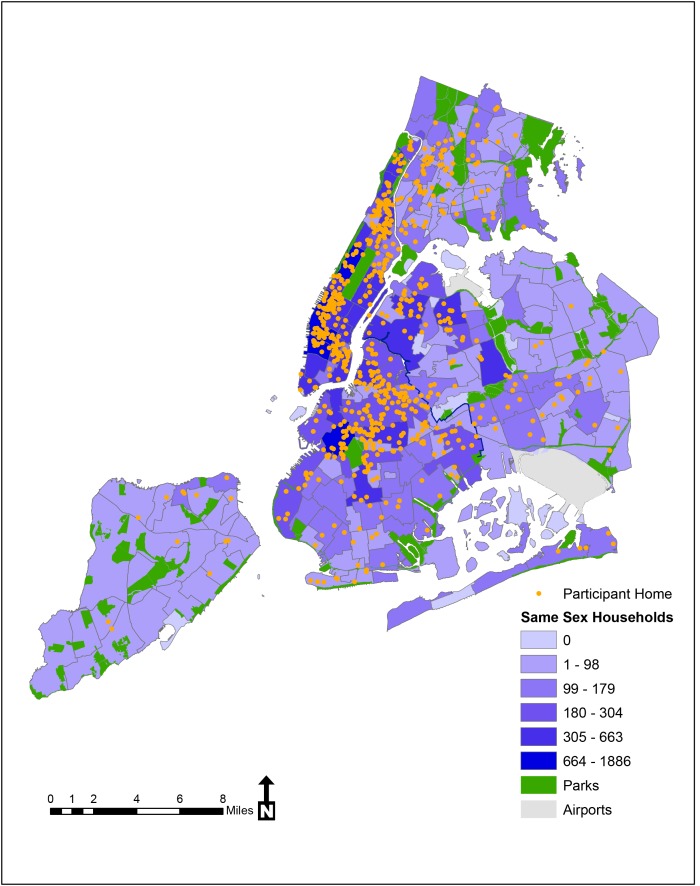
Home neighborhoods overlaid on same sex headed-households (2010 US Census data), NYCM2M Study.

Figure 1 shows the participant home locations overlaid on the number of Census reported same sex headed-households. The distribution of residences of participants is dispersed throughout the city with concentrations in some areas with higher same sex headed-households, while other areas of high same sex headed-households have a lower concentration of participants. Most importantly, the figure illustrates how the role of one neighborhood level covariate (proportion of same sex headed-households) can be utilized to examine its role in sexual risk behavior, substance use, and mental health among urban MSM.

**Figure 2:**
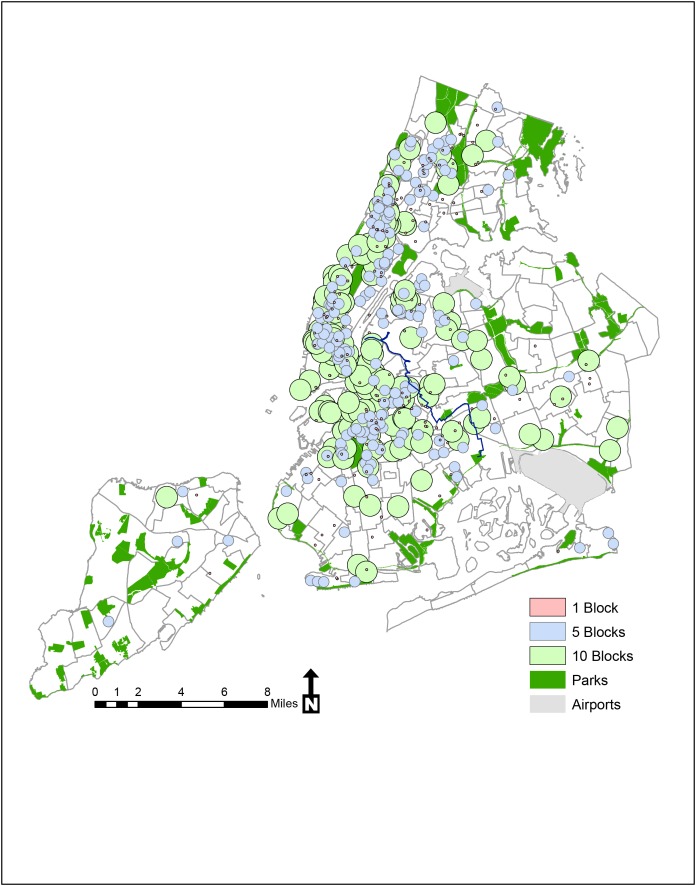
Participant-described home neighborhood boundaries, NYCM2M Study.

Figure 2 provides an illustration of how individual men conceived of neighborhood based on their perceived geographical residential neighborhood and represents an initial step in incorporating participant-defined neighborhoods. Using these data, we calculated 1 block (dots), 5 block (blue circles), and 10 block (green circles) buffers (using an estimate of 0.059 miles/block) to describe each participant’s neighborhood.

**Social Neighborhood.** Men aggregated in fewer social neighborhoods with only 89 (26%) of the 347 neighborhoods represented. In terms of the boundaries of these social neighborhoods, the plurality of men (36%) reported that their social neighborhoods spanned areas larger than 10 blocks around the pin drop; another 31% reported that it was the area within 10 blocks; 20% said it was within 5 blocks and 14% said it was a specific block. The majority of men (87%) reported having no relatives living in their self-identified social neighborhoods. Thirty-one percent reported having no friends who lived in their social neighborhood. Sixty percent reported having no sexual partners who lived in their social neighborhoods.

**Sexual Neighborhood.** Participants identified 127 (37%) different neighborhoods as a recent or most often sexual neighborhood. Sexual neighborhoods were smallest with most men (73% most often; 75% most recent), describing an area five blocks or less.

**Congruence of Neighborhoods.** *:* A minority of men (15%) reported congruence of home, social and sexual neighborhoods. Thirty-one percent of men reported that none of their neighborhoods were the same. For 39% of men, their home and sexual neighborhoods were the same, but social neighborhood was different and 10% of men reported that their social and sexual neighborhoods were the same (with home being different). Only 4% of men reported that their home and social neighborhoods were the same, with sexual neighborhoods different. Congruence of neighborhoods differed significantly by race/ethnicity (p = 0.0129). A higher percent of White men (22%) reported that all of the neighorboods were the same, compared to Black (10%) and Latino (11%) and a higher percent of Black (35%) and Latino (37%) reported that none of their neighborhoods were the same compared to White men (25%). Among men who reported that their home, social and sexual neighborhoods were not the same neighborhood, less than 3% defined all their neighborhoods as the same size.

## Discussion

The methodology and selected results described here illustrates the potential that innovative spatial analytic methods offer to characterize multiple influential contexts for MSM in the urban environment and addresses many of the limitations that characterize neighborhood effects research. Our previous research [20], [21], [53] provided initial insights into neighborhood typologies and functions, allowing us to map multiple neighborhoods of potential influence: “home” “social” and “sexual”. We hypothesize that in addition to where men live, they are also influenced by the environments in which they socialize and have sex as well as the duration and intensity of exposure to neighborhood environments.

Our use of Google Earth as a method of neighborhood data collection allowed us to characterize the location and scale of home, social and sexual neighborhoods of a diverse sample of MSM in NYC. We were able to collect specific locations (pin drops) in a secure manner from which we could then construct participant-defined or uniquely buffered neighborhood boundaries rather than relying only on predetermined administrative boundaries (e.g. zip code, census tract) for home, social and sexual neighborhood spaces. The Google Earth method of collected geocoded locations also reduced error that may be introduced if street intersections are manually recorded, with the potential of typographical errors, misspelling and abbreviations which can result in difficulty in geocoding. Rather than using administrative boundaries to define neighborhoods, we allowed study participants to provide their own definitions of the size of influential space in these three contexts. Our approach to assessing neighborhood scale allows us to conduct analyses that use neighborhood boundaries that are most appropriate for specific outcomes, as well to conduct sensitivity analyses that will evaluate whether and how use of various boundaries alters relations among neighborhood covariates and selected outcomes.

Our initial results reported here revealed that men perceived their home, social and sexual neighborhoods in different ways. Of the three neighborhood types, the number of unique home neighborhoods was the largest and unique social neighborhoods was the lowest. Men in the study sample underrepresented men 18 years of age and older in three of the four outer boroughs of NYC. Further analyses will be conducted to assess whether addition of the internet and mobile application recruitment strategy helped to expand the number of home, social and sexual neighborhoods identified. Men also identified their social neighborhoods as being the largest while the size of their sexual neighborhoods was the smallest. Such information could be informative, for example, in the analysis of community viral load by neighborhoods. Few men (15%) had the same home, social and sexual neighborhoods while 31% indicated that none of the three neighborhoods was the same. Significant differences were found in congruence of neighborhoods by race/ethnicity with men of color reporting more separation of their neighborhoods, potentially reflecting differences in socioeconomic status and/or experiences of stigma in certain neighborhoods. [21]

The methods applied in this study have resulted in a unique and unprecedented dataset allowing us to test hypothesized relations among the multiple neighborhood social and physical environments that MSM inhabit and MSM’s sexual behavior and health. This multi-dimensional neighborhood-level database includes social environmental factors, such as collective efficacy, physical environmental factors, such as green space, and compositional factors relevant to MSM, such as same sex headed- households and other indicators of “gay presence.” Together these datasets reflecting both perceived neighborhood characteristics (subjective neighborhood conditions), as well as merged data from numerous archival sources (objective neighborhood conditions) create a flexible and rich neighborhood-level set of covariates. For example, we will be able examine whether neighborhood-level attitudinal norms around sexual risk behaviors are related to sexual risk behavior, while controlling for individual-level factors. Drawing upon social identity theory, we can evaluate whether this association is moderated by an individual’s level of attachment to and time spent in residential neighborhood and social identity. We can evaluate whether this relationship holds true for men who reside in the neighborhood and men who spend most of their social time in the neighborhood. As another example, drawing upon sexual minority stress theory, we can examine the influence of perceived neighborhood-level homophobia on men who do not reside in gay enclaves. It is possible that men who live in neighborhoods with perceived high levels of perceived homophobia will have reduced opportunities for sex and thus will be lower risk simply for this reason. However, this reduced risk due to reduced opportunity may be off-set by increased risk associated with traveling to a gay enclave to find sex partners, and potentially the stress associated with the daily experience of homophobia and not being exposed to safer sex attitudinal norms of gay enclaves. This line of research is particularly important, as many minority men in NYC are unable to afford the housing costs associated with living in a gay enclave. Our investigation of the home, social and sexual neighborhoods is a first step to more accurately measure the impact of these environments on individuals. Advancing this knowledge is critical to neighborhood effects on health research base and informs the continued debate of what neighborhood means and to whom.

There are several limitations to the study methods and resultant data that should be noted. While venue-based time-space sampling is a highly developed method for the recruitment of hard to reach or hidden populations, the sample may over represent men more engaged with bar and club culture. Several steps were taken to limit this effect such as including a broad array of venues not associated with bar/clubs and the use of gay networking mobile apps. Furthermore, the men recruited self-select to participate likely having an impact on the sample composition. While these data are not generalizable to other cities, the methods described here can be used in other places to analyze local effects on MSM health. The study is cross-sectional which limits our ability to consider how home, social, and sexual neighborhoods change over time, although length of residence was assessed. The map of buffers around home neighborhoods does not reflect topographical barriers, such as the Hudson River, and does not account for differences in block size in different areas of NYC. Finally, although we will use archival data for neighborhood-level characteristics, we are reliant on self-report data on outcomes and several key neighborhood-level covariates, such as neighborhood homophobia.

Many studies have described the biological and social factors that contribute to the health disparities experienced by MSM including elevated rates of HIV and STIs [1], [54], [55] and higher rates of mental health (e.g. depression, anxiety) and substance use disorders [56]–[60]. In a recent Lancet paper, Mayer et al. argue that the particular experiences and needs of MSM require a comprehensive and holistic view towards MSM health and MSM-related research [61]. The methods described here will hopefully provide guidance for future studies on neighborhoods and MSM health, as well as other types of neighborhood studies. Our study will provide important groundwork for the development of structural and neighborhood-based interventions, as well as for identifying approaches that augment individual-level interventions through community development initiatives and health messages for MSM specific to their neighborhood context.
